# Establishment of ITS-Derived Species-Specific PCR Assay Method for Discriminating Herbal Medicine Descurainiae Semen from Its Commercial Adulterants

**DOI:** 10.3390/plants15010073

**Published:** 2025-12-25

**Authors:** Wook Jin Kim, Sungyu Yang, Woojong Jang, Byeong Cheol Moon

**Affiliations:** Herbal Medicine Resources Research Center, Korea Institute of Oriental Medicine, Naju 58245, Republic of Korea; ukgene@kiom.re.kr (W.J.K.); sgyang81@kiom.re.kr (S.Y.); wjjang@kiom.re.kr (W.J.)

**Keywords:** genetic authentication, herbal medicine, industrial material, Descurainiae Semen, rDNA-ITS, SCAR-PCR assay

## Abstract

Accurate authentication of herbal medicine Descurainiae Semen, the tiny seeds of *Descurainia sophia*, is challenging due to their morphological similarity to various adulterants. To develop a precise and reliable molecular identification method, we conducted comparative analyses of rDNA-ITS sequences using *D. sophia* and five adulterant species and subsequently developed species-specific sequence-characterized amplified region (SCAR) markers. The discriminatory power and detection limits of these markers were evaluated using serially diluted genomic DNA from each species and commercially available Descurainiae Semen, respectively. The SCAR markers developed in this study enabled the detection of adulterant contamination at levels as low as 0.01–1%. Among several potential adulterants tested using 17 herbal medicines, *Erysimum macilentum* was found to be the most common adulterant in commercial products, with a ratio of 88%. The SCAR-PCR assay established in this study provides a rapid and accurate tool for identifying *D. sophia* and illegal adulterants at the species level and at very low contamination levels, thereby supporting improved quality control and enhancing consumer confidence in the herbal medicine industry.

## 1. Introduction

*Descurainia sophia* (L.) Webb ex Prantl is a medicinal plant extensively used not only in traditional medicine but also in food, beverages, cosmetics, and health-related industries [1,2,3]. Its seeds are used to make the traditional beverage Khak-e Shir [4] and are widely prescribed in East Asian traditional medicine for respiratory diseases such as asthma and bronchitis. However, inconsistent definitions of botanical origin across national pharmacopeias have led to ambiguity in the standardization, quality control, and appropriate use of these medicinal seeds. The Korean Herbal Pharmacopoeia (KHP) lists both *D. sophia* and *Lepidium apetalum* Willd. as authentic species of Lepidii seu Descurainiae Semen (LDS), called “*Jeong-Lyeok-Ja*” in Korean; however, the Pharmacopoeia of the People’s Republic of China (ChP 2015) separately lists the authentic species of Descurainiae Semen (DS) as *D. sophia*, and the authentic species of Lepidii Semen (LS) as *L. apetalum* [5]. These differences are not just taxonomic; they are closely related to their specific therapeutic uses. DS is primarily prescribed for respiratory disease, while LS is less commonly used and mainly employed as a diuretic [2,6]. Compounding this issue, the availability and authenticity of these medicinal materials are further complicated by regional flora. *D. sophia* is native to Korea, whereas *L. apetalum* is not; nevertheless, several *Lepidium* species have been introduced and domesticated. Morphologically similar species such as *Lepidium densiflorum* Schrad. and *Lepidium virginicum* L. are likely to be misused as adulterants of LS, while *Erysimum cheiranthoides* L. and *Erysimum macilentum* Bunge have been reported as adulterants of DS due to their resemblance to *D. sophia* [7,8]. Moreover, confusion regarding the scope of authentic species persists, as *Draba nemorosa* L., previously listed as an authentic source in the KHP, was excluded in the 2013 version, further highlighting inconsistencies in pharmacopeial standards [5].

Given the expanding use of these seeds as raw materials for dietary supplements in addition to traditional medicines, the presence of such taxonomic uncertainty and frequent adulteration poses serious risks to product efficacy, safety, and regulatory compliance [9,10]. Therefore, accurate authentication of the botanical origins of pharmacopeial herbal medicines and dietary supplemental ingredients is critically important, underscoring the need for reliable, standardized identification methods to prevent adulteration and ensure quality control.

DNA barcoding is one of the most widely used and fundamental approaches for genetic discrimination at the plant species level [11,12,13]. However, the routine implementation of this approach in quality control is limited by the need for individual-level DNA sequencing, downstream bioinformatic analyses, and genomic DNA of adequate quality to ensure reliable amplification. Recent studies have highlighted both the potential and limitations of DNA barcoding for authenticating commercial products, particularly in bulk or mixed samples [11,12].

Advanced molecular diagnostic approaches, such as quantitative PCR (qPCR) and DNA metabarcoding, have also been proposed as effective alternatives for detecting adulterants and species diversity in commercial products; however, their practical implementation remains constrained by high analytical costs and the need for specialized infrastructure [14,15]. In contrast, species identification based on conventional PCR can be readily applied to routine authentication without sequencing, offering a rapid, cost-effective alternative. Consequently, conventional PCR-based assays have been widely used as simple, practical tools for the genetic authentication of medicinal plant resources and food raw materials [16,17,18].

The sequence-characterized amplified region (SCAR) marker-based PCR assay is a simple, inexpensive, and widely used method for identifying various natural products, including medicinal plants; indeed, it is much simpler than DNA barcoding [19,20]. In addition, sample analysis is very convenient because seeds are analyzed in bulk rather than individually; other advantages include the ability to confirm the approximate mixing ratio by measuring the density of the PCR products [17,21]. The ribosomal DNA-internal transcribed spacer (rDNA-ITS) region targeted in this assay and applied universally to obtain species-specific nucleotide (SSN) sequences, exhibits high interspecies sequence variation, making it very useful for DNA barcoding [11,13,17].

To address the shortcomings mentioned above, the present study used sequences discriminating rDNA-ITS regions between *D. sophia* and adulterant species with high morphological similarity to obtain SSN sequences. SCAR-PCR assays were then conducted, resulting in more efficient and rapid identification of species than DNA barcoding. The SCAR-PCR assay allows species-specific primer design based on SSN sequences. In addition, by analyzing commercial LDS herbal products distributed in markets, we demonstrated that the SCAR-PCR assays are a practical and reliable diagnostic tool that could be used by regulatory institutes, and by industrial companies to improve quality control and product safety.

## 2. Results

### 2.1. Sequence Analysis of the rDNA-ITS Region

The rDNA-ITS region from all 27 specimens (four *D. sophia*, three *L. densiflorum*, seven *L. virginicum*, three *E. cheiranthoides*, four *E. macilentum*, and six *D. nemorosa*) was successfully amplified. The resulting amplicons were cloned into a T-vector plasmid and sequenced using the SP6 primer. The results revealed that all six species produced sequences of uniform length within each species: 710 bp for *D. sophia*, 709 bp for *L. densiflorum*, 709 bp for *L. virginicum*, 707 bp for *E. cheiranthoides*, 708 bp for *E. macilentum*, and 705 bp for *D. nemorosa*, respectively (Figure S1). To verify the identity of the 27 morphologically identified specimens, the nucleotide sequences were searched using the Basic Local Alignment Search Tool (BLAST, https://blast.ncbi.nlm.nih.gov/Blast.cgi, accessed on 25 December 2025) on the National Centre for Biotechnology Information (NCBI) platform. The results confirmed that the sequences were consistent with the morphological identification (Table 1).

### 2.2. Phylogenetic Analysis

**Figure 1:**
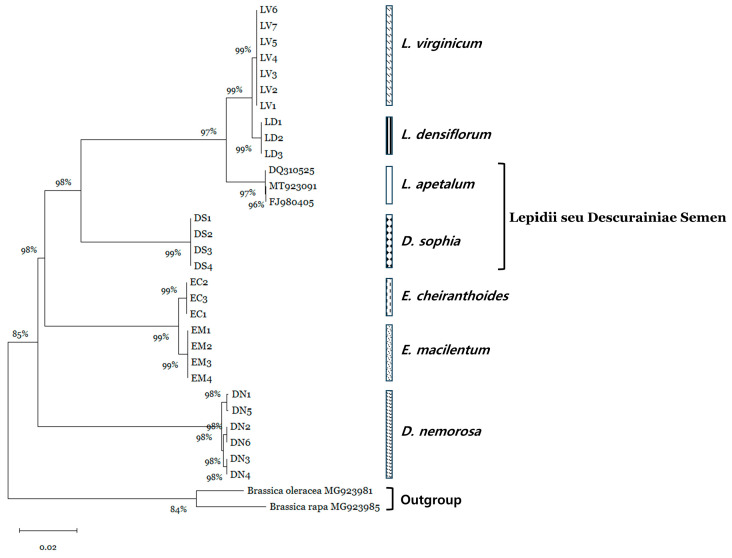
Phylogenetic analysis of six species. The phylogenetic tree was reconstructed using the neighbor-joining (NJ) method based on the rDNA-ITS sequences of 32 accessions, including 27 specimens representing six species and three *L. apetalum* sequences retrieved from NCBI GenBank. Two *Brassica* species were used as the outgroup.

Phylogenetic analysis based on the rDNA-ITS sequences was conducted for 30 specimens derived from seven species, including additional 3 accessions of *L. apetalum*, an authentic species of Lepidii Semen listed in both the KHP and ChP 2015 (Figure 1). Neighbor-joining (NJ) analysis revealed that each species formed a monophyletic clade. *L. virginicum*, *L. densiflorum*, and *L. apetalum*, all belong to the genus *Lepidium*, formed a sister group, as did *E. cheiranthoides* and *E. macilentum*, which belong to the genus *Erysimum*. With the exception of *D. nemorosa*, in which intraspecies nucleotide variation was confirmed, the five species sequenced in this study showed no intraspecies nucleotide variation, indicating a strong phylogenetic relationship.

### 2.3. Species-Specific Primer Design and Establishment of Optimal PCR Conditions

**Figure 2:**
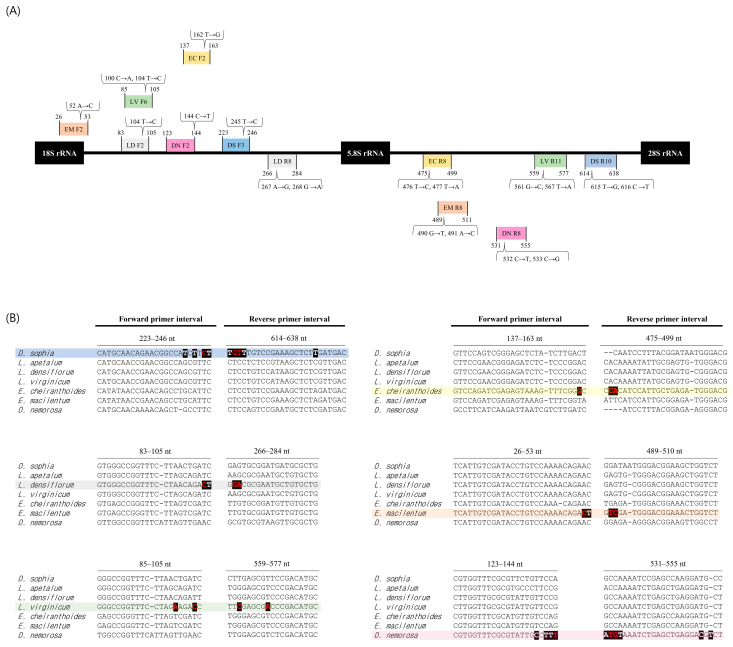
Comparative analysis of the rDNA-ITS region and the positions of the designed SCAR primers -in, including nucleotide variations in designed SCAR primers. (**A**) The positions of the designed SCAR primer are indicated by the colored boxes. Brackets above or below the boxes denote the arbitrarily substituted nucleotides, with their corresponding position numbers. (**B**) The highlighted colored text represents nucleotides within the SCAR primer interval designed for each species, with the bold red letters indicating arbitrarily substituted nucleotides. Bold white text in black shading indicates nucleotides that are species-specific compared to the other six species.

**Figure 3:**
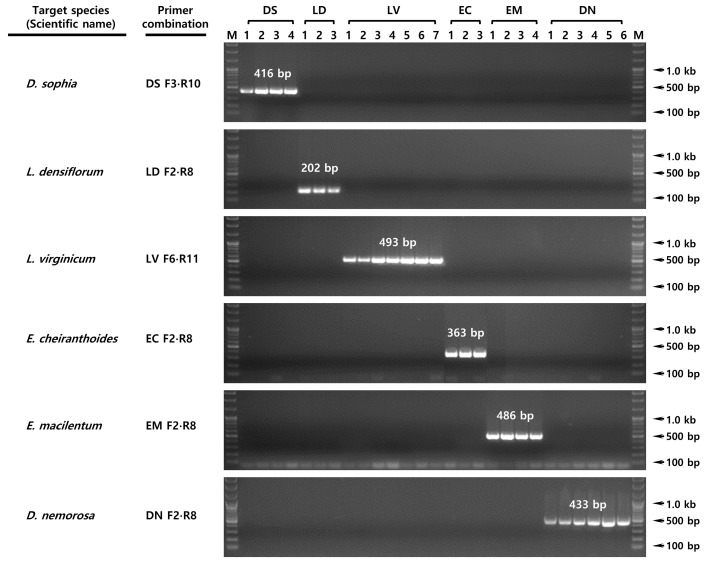
Testing the specificity of the species-specific SCAR primers using conventional PCR. Each electrophoresis image shows the amplicons from the 27 specimens generated respective species-specific SCAR primer sets. Sample information is provided in Table 1. DS, *D. sophia*; LD, *L. densiflorum*; LV, *L. virginicum*; EC, *E. cheriranthoides*; EM, *E. macilentum*; DN, *D. nemorosa*. M, 100 bp DNA ladder. The target species and the corresponding primer combinations used for the species-specific SCAR-PCR assays are indicated on the left.

The 30 rDNA-ITS sequences were aligned based on maximum nucleotide identity using ClustalW, and SCAR primers (including those with an SSN sequence located at the 3′ end) were selected (Figure 2 and Figure S2). To increase specificity, each species was also analyzed using forward and reverse primers containing arbitrary nucleotide substitutions. *D. sophia* was subjected to SCAR using the DS F3 and R10 primer pair, a species-specific DNA fragment of 416 bp was amplified (Figure 3). Similarly, primers LD F2 and R8 yielded a species-specific DNA fragment of 202 bp for *L. densiflorum*, LV F6 and R11 yielded 493 bp for *L. virginicum*, EC F2 and R8 yielded 363 bp for *E. cheiranthoides*, EM F2 and R8 yielded 486 bp for *E. macilentum*, and DN F2 and R8 yielded 433 bp for *D. nemorosa,* respectively. The optimal PCR conditions for each SCAR assay were confirmed by gradient PCR using these species-specific primer combinations, and the optimal annealing temperature was determined 63 °C, except for *E. cheiranthoides*, which required an annealing temperature of 67 °C.

### 2.4. Evaluation of Sensitivity and Adulterant Context in Commercial Products

**Figure 4:**
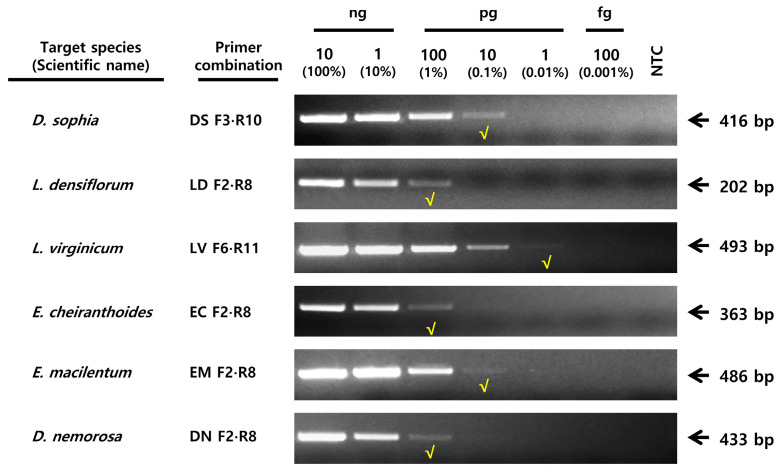
Determination of the LOD of the species-specific SCAR primers using conventional PCR. Each electrophoresis image shows the detection limits obtained with serially diluted DNA and the corresponding SCAR primer sets. M, 100 bp DNA ladder; NTC, no template control. The target species and the primer combinations used for the species-specific SCAR-PCR assays are indicated on the left. The check (√) symbol denotes the amount of template DNA corresponding to the LOD.

**Figure 5:**
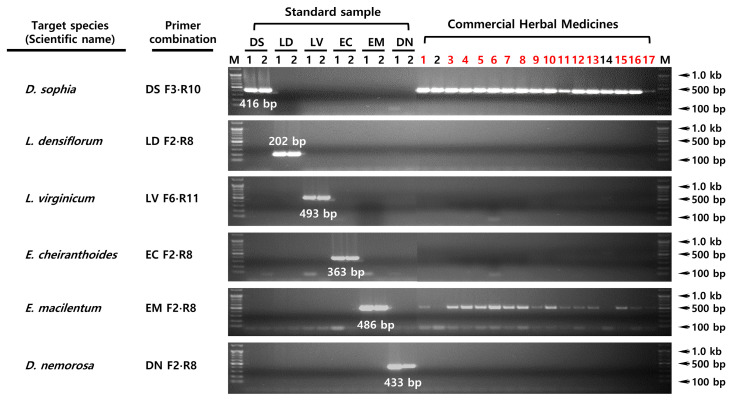
SCAR-PCR assay of commercial products. The results show the extent to which 17 commercial products sold in the herbal market are contaminated with other species, as detected by SCAR PCR. Standard samples used in this experiment are listed in Table 1, and the commercial LDS/DS products are listed in Table S1. M, 100 bp DNA ladder. The target species and corresponding primer combinations used for the species-specific SCAR-PCR assays are indicated on the left. Red-numbered commercial LDS/DS products denote those adulterated with the counterfeit species *E. macilentum*.

The sensitivity test was performed under optimal PCR conditions using primers with confirmed specificity. The results showed that the LOD values differed among the six species tested. *D. sophia* and *E. macilentum* were detectable by conventional PCR at 10 pg of DNA corresponding to an LOD of 0.1%, whereas *L. virginicum* was detectable at 1 pg, corresponding to an LOD of 0.01% (Figure 4). In contrast, *L. densiflorum*, *E. cheiranthoides*, and *D. nemorosa* were detectable to 100 pg (LOD, 1.0%). Next, we applied SCAR PCR assay to 17 commercial LDS products distributed in the Korean and Chinese herbal markets (Figure 5, Table S1). The results revealed that 15 of these products contained authentic *D. sophia* adulterated with *E. macilentum*. Only two products contained “pure” *D. sophia* (i.e., no *E. macilentum* was detected at 10 pg DNA, corresponding to an LOD of 0.1%). No other potential adulterant species were detected.

## 3. Discussion

The motivation for this study arose from the discovery of LDS adulterants during a pervious investigation accessing the anticancer efficacy of commercially available LDS herbal medicines in Korea. During the long experimental process, we observed that LDS extracts exhibited variable anticancer activities depending on the lot numbers of herbal medicines. Thus, anticancer activity of LDS was different depending on the lot number with geographical regions of cultivation or years (unpublished data). These findings suggest that diverse factors such as harvesting time, geographical origin, and batch-to-batch variation may significantly influence the bioactivity of LDS, highlighting the importance of accurate species authentication and quality control in herbal medicine research and development. To clarify the origin of authentic species of these LDS, we analyzed the morphology of individual seeds of commercial LDSs introduced for anticancer activity under a microscope, and identified significant morphological differences [8]. To further explore these differences, we classified the seeds based on morphology and then analyzed DNA barcode sequences. Comparing the DNA barcode sequences with those obtained from NCBI GenBank BLAST analysis revealed that most of the commercial LDSs contained seeds belonging to the genus *Erysimum* [7]. To identify the species of the adulterant seeds, we collected *E. cheiranthoides* and *E. macilentum*, along with other LDS-related plant species in Korea (Table 1), and performed further DNA barcode analyses. The results confirmed that the seeds adulterating commercial LDSs were *E. macilentum* [7,8].

However, despite the clear identification of the adulterant species, practical approaches for routinely authenticating *D. sophia* in commercial LDS products remain limited. Although *D. sophia* is in high demand because of its antioxidant and anti-obesity properties, its misuse is a serious concern due to morphological similarities with other species and revisions to pharmacopeia standards [22,23]. Despite its widespread use and the risk of adulteration, there is no molecular method for authenticating *D. sophia* or its commercial adulterants; only visual and organoleptic discrimination, including morphological analysis and DNA barcoding of the *mat*K region, are used at present [7,8]. It is possible to distinguish species using visual and organoleptic methods, as well as microscopic characteristics; however, *D. sophia* seeds are tiny, measuring 0.8–1.2 mm long × 0.5 mm wide, and thus require analysis under a microscope [7,8]. This method is limited in its ability to confirm the degree of adulteration. Identification of LDS species using sequence analysis of the *mat*K region [7], a genetic analysis method based on DNA barcoding [24], can be conducted, but it is cumbersome because it requires a series of experimental steps (PCR amplification, gel rescue, and sequence analysis). In addition, accurate species identification is only possible when the sequence of each individual seed is analyzed, although this is not easy when the seeds are tiny.

Therefore, the aim of the present study was to develop a simple and reliable tool that enables genetic differentiation tool of *E. sophia*, which is used as an herbal medicine and has potential applications in food, cosmetics, agriculture, and environmental fields [1,2,3], from other adulterating species. To achieve this, we analyzed the rDNA-ITS region sequences, a universal DNA barcode commonly used for species-level identification, in 27 specimens selected and identified based on botanical morphology. Phylogenetic analysis revealed that all six species identified from these specimens formed a monophyletic clade, proving that the corresponding regions are an effective DNA barcode for developing a genetic diagnostic method (Figure 1). Furthermore, the phylogenetic relationships observed for some species in this study are consistent with those reported by Li et al. [25], who assessed phylogeny of LDS-related plants based on chloroplast genome analysis.

Based on multiple sequence alignment analysis, we designed six sets of primers, each containing SSN sequences at the 3′ end and containing arbitrary nucleotide substitutions (Figure 2). These primers were then used to develop an optimal species-specific PCR assay (Figure 3). Because we were unable to collect samples of *L. apetalum*, an authentic species used as LDS along with *D. sophia*, development of a genetic diagnosis method for this species was not possible; however, multiple alignment of *L. apetalum* sequences registered in the NCBI GenBank confirmed the presence of SSN sequences. Therefore, if samples of *L. apetalum* are secured in the future, we expect to develop a diagnostic PCR for this species (Figure S1). Furthermore, we demonstrated that the conventional PCR assay using SCAR primers was specific for *L. densiflorum* and *L. virginicum*, which are close relatives of *L. apetalum* (Figure 3). Multiple alignment analysis showed that the intervals between the primer designed for two species were species-specific (Figure 2B); therefore, we predict that the possibility of misidentifying *L. apetalum* as one of these closely related species, albeit indirectly, is very low [17,21].

To evaluate the discriminatory ability of each SCAR-PCR primer sets, we tested a range of commercial products. We found that 15 of 17 products contained *D. sophia* adulterated with *E. macilentum*, confirming that most of LDS products distributed in Korea and China are impure (Figure 5, Table S1). These results indicate that adulterant species are being mixed with *D. sophia* and sold as LDS. The proportion of adulterants in many commercial products is sufficiently high to warrant attention as a quality control issue by appropriate regulatory authorities. In addition, we confirmed that the established SCAR assay could detect within the range of 1–100 pg (0.01–1.0%) in the LOD assay. According to the KHP, impurities or adulterants are generally permitted at levels of 1.0–2.0%. LOD values of the established SCAR assay are more sensitive to than those reported for conventional PCR [16,17,26].

Nevertheless, the SCAR-PCR assay developed in this study has several limitations. First, although the assay exhibits high species specificity and sensitivity, it is a qualitative method. It does not provide precise quantitative information on adulterant levels, unlike qPCR [27]. Second, unlike qPCR-based assays, which can be configured for multiplex detection using various probes and amplicon sizes, the SCAR-PCR assay can potentially enable single-species discrimination, as developed in this study [28,29]. Third, this assay’s performance was not evaluated for highly processed commercial products subjected to intensive thermal or chemical treatments, which may degrade the DNA and affect amplification efficiency. Therefore, there remains a need to develop advanced analytical methods capable of accurately quantifying adulterant levels and simultaneously discriminating multiple species in DS, a medicinal and food-related resource of high commercial value. Despite these limitations, the SCAR-PCR assay presented here demonstrates clear practical advantages, not in terms of methodological novelty, but in its simplicity, cost-effectiveness, and applicability to large-scale commercial samples.

Therefore, the SCAR assay developed in this study demonstrates high sensitivity suitable for the purity testing of herbal medicines, and is expected to contribute to the quality control of herbal medicines and the assurance of consistent pharmaceutical efficacy. The SCAR PCR assay described herein is an accurate and cost-effective method for assessing the quality of very tiny sized *D. sophia* seed, confirming the presence of adulterants in commercial products and thereby revealing quality issues with DS distributed in the market. Furthermore, this simple assay can be readily applied by relevant regulatory institute, making it a useful tool for detecting serious quality problems caused by adulteration in DS products.

## 4. Materials and Methods

### 4.1. Plant Material and Morphological Identification

A total of 27 specimens, representing six species (*D. sophia*, *L. densiflorum*, *L. virginicum*, *E. cheiranthoides*, *E. macilentum*, and *D. nemorosa*), were collected from Korea and China for this study (Table 1, Figure S3). All specimens, identified by plant taxonomists and herbologists, were deposited in the Korean Herbarium of Standard Herbal Resources (Index Herbariorum [IH] code: KIOM).

### 4.2. Extraction and Quantification of Genomic DNA

Leaf tissue (100 mg) was added to 1.5 mL of AP1 buffer in a Lysing Matrix A tube (mpbio, Irvine, CA, USA) and ground (5000 rpm for 30 s) using a Precellys™ Grinder (Bertin Technologies, Montigny-le-Bretonneux, France). Genomic DNA was extracted using the DNeasy Plant Mini Kit (Qiagen, Valencia, CA, USA), as described by Kim et al. [20]. The quantity of genomic DNA was measured using an ND-1000 UV/Vis spectrophotometer (NanoDrop, Wilmington, DE, USA). Genomic DNA was diluted to a concentration of 10–20 ng/μL prior to use for rDNA-ITS amplification, SCAR specificity and sensitivity testing, and evaluation of adulterants in commercial products.

### 4.3. Sequence Analysis of the rDNA-ITS Region

PCR amplification was performed using universal primers ITS1 (5′-TCC GTA GGT GAA CCT GCG G-3′) and ITS4 (5′-TCC TCC GCT TAT TGA TAT GC-3′) targeting the rDNA-ITS region [30]. PCR mixtures included 0.5 μM of the ITS1 and ITS4 primers, Solg™ 2×Taq PCR Smart-Mix I (Solgent, Daejeon, Korea), and template DNA (10–20 ng) in a total reaction volume of 40 μL. The PCR reactions were performed in a Proflex PCR system (Applied Biosystems, Foster City, CA, USA) under the following conditions: initial denaturation at 95 °C for 2 min, followed by 35 cycles of denaturation at 95 °C for 40 s, annealing at 53 °C for 40 s, and extension at 72 °C for 1 min, with a final extension at 72 °C for 5 min. Each PCR product was analyzed by electrophoresis (150 V for 40 min) in 1.5% agarose gels, with a 100 bp DNA ladder (Solgent, Daejeon, Korea). The expected band of 600–700 bp was recovered using a Gel Extraction Kit (Qiagen, Valencia, CA, USA). After gel extraction, we conducted T-vector ligation and transformed *E. coli* to obtain the complete sequence of the amplified rDNA-ITS region. All following experiments (i.e., T-vector ligation, *E. coli* transformation, colony PCR, Sanger sequencing, and sequence analysis) were carried out as described by Kim et al. [20].

### 4.4. Phylogenetic Analysis

Phylogenetic analysis of the rDNA-ITS sequences was performed using MEGA version 6.06 software [31]. A phylogenetic tree was reconstructed using the neighbor-joining (NJ) method and the K2P model, pairwise deletion to address gaps/missing data, and 1000 bootstrap replications, as described by Kim et al. [20]. The rDNA-ITS sequences from 27 specimens were analyzed together with three GeneBank-registered accessions of *L. apetalum* (DQ310525, MT923091, and FJ980405). *Brassica oleracea* (MG923981) and *B. rapa* (MG923985) were included as outgroup.

### 4.5. Species-Specific Primer Design and Specificity Testing

Species-specific primers were designed to encompass nucleotide sequence variations identified through maximum alignment of sequences obtained from ITS1–ITS4 primer amplification of 27 accessions from six species using the BioEdit program (version 7.0.5.3). We specifically designed the SCAR primers to target species where SSNs or nucleotides with low homology to other species were located at the 3′ ends. For the SCAR, species-specific primers were designed to generate small amplicons of <500 bp in length. In addition, an arbitrary nucleotide substitution was incorporated into the forward and/or reverse primers (Table 2). Primers were used in four combinations to test specificity, using forward and reverse primers containing unmodified nucleotides as well as those with arbitrary substitutions. The PCR reactions comprised 0.25 µM of each primer, Solg™ 2×Taq PCR Smart-Mix I (Solgent, Daejeon, Korea), and 10 ng of genomic DNA in a total volume of 20 µL. Optimal PCR annealing temperatures ranged from 55 to 67 °C. The amplification conditions were as follows: initial denaturation at 95 °C for 2 min; 35 cycles of denaturation at 95 °C for 30 s, annealing for 30 s, and extension at 72 °C for 30 s; followed by a final extension step at 72 °C for 5 min.

### 4.6. Determination of the Limit of Detection (LOD)

The sensitivity of each species-specific primer pair was assessed by amplification of genomic DNA, serially diluted ten-fold from 10 ng (100%) to 100 fg (0.001%). All experiments were conducted in triplicate, using the selected species-specific primer pairs under optimal PCR conditions. The detailed experimental protocols are described by Kim et al. [20].

### 4.7. Evaluation of Adulterants in Commercial Products

Commercial products distributed in the Korean and Chinese herbal markets were evaluated by conventional PCR to confirm whether adulterants were mixed with the authentic species (Table S1). For these analyses, approximately 1 g of material—equivalent to more than 300 randomly selected seeds—from each commercial product was finely ground using a grinder. Subsequently, 100 mg of the resulting powder was used for genomic DNA extraction. The extraction of genomic DNA and the SCAR-PCR assay were carried out as described in Section 4.2 and Section 4.5 above.

## 5. Conclusions

We developed species-specific SCAR markers for Descurainiae Semen based on rDNA-ITS sequence analysis, providing a rapid and reliable method for authenticating this medicinal seed and detecting morphologically similar adulterants. These markers exhibited high sensitivity, allowing for the detection of contaminations as low as 0.01–1%. Application of the SCAR-PCR assay to commercial herbal medicine products identified *E. macilentum* as the most frequent adulterant. This SCAR-PCR assay offers a practical tool for ensuring the quality, purity and authenticity of Descurainiae Semen, thereby supporting improved quality control, regulatory compliance, and consumer confidence in the herbal medicine industry.

## Figures and Tables

**Table 1:** List of specimens with collection details and GenBank accession numbers.

Herbal Name	Scientific Name	Voucher	Abbr.	Collection Site	GenBank
Descurainiae Semen	*Descurainia sophia* (L.) Webb ex Prantl	CNU #55	DS1	Hwaksan, Goro, Gunwi, Korea	PX313848
		MBC_KIOM-2016-8	DS2	Gangseo 2, Heungdeok, Cheongju, Korea	PX313849
		MBC_KIOM-2016-13	DS3	Bisan, Soi, Eumseong, Korea	PX313850
		MBC_KIOM-2016-38	DS4	Chubu, Geumsan, Korea	PX313851
-	*Lepidium densiflorum* Schrad.	MBC_KIOM-2016-88 #1	LD1	Myeongwol, Ando County, Jilin Province, China	PX313852
		MBC_KIOM-2016-88 #2	LD2	Myeongwol, Ando County, Jilin Province, China	PX313853
		MBC_KIOM-2016-88 #3	LD3	Myeongwol, Ando County, Jilin Province, China	PX313854
	*Lepidium virginicum* L.	CNU #32	LV1	Jeongbyeongsan, Uichang, Changwon, Korea	PX313855
		CNU #156	LV2	Hwaksan, Goro, Gunwi, Korea	PX313856
		MBC_KIOM-2016-23	LV3	Miho, Daedeok, Daejeon, Korea	PX313857
		KWJ_KIOM-2016-2	LV4	Goeso, Samgi, Gokseong, Korea	PX313858
		YSG_KIOM-2016-15	LV5	Songgye, Jecheon, Korea	PX313859
		MBC_KIOM-2016-26	LV6	Sinan, Sancheong, Korea	PX313860
		MBC_KIOM-2016-52	LV7	Anmyeondo, Taean, Korea	PX313861
	*Erysimum cheiranthoides* L.	MBC_KIOM-2016-228 #1	EC1	Jeoksang, Danyang, Korea	PX313862
		MBC_KIOM-2016-228 #2	EC2	Jeoksang, Danyang, Korea	PX313863
		MBC_KIOM-2016-228 #3	EC3	Jeoksang, Danyang, Korea	PX313864
	*Erysimum macilentum* Bunge	MBC_KIOM-2016-40 #1	EM1	Sinwol, Gurye, Korea	PX313865
		MBC_KIOM-2016-40 #2	EM2	Sinwol, Gurye, Korea	PX313866
		CGY_KIOM-2016-8 #1	EM3	Pungcheon, Andong, Korea	PX313867
		CGY_KIOM-2016-8 #2	EM4	Pungcheon, Andong, Korea	PX313868
	*Draba* *nemorosa* L.	CNU #174	DN1	Janggunbong, Bonghwa, Korea	PX313869
		YSG_KIOM-2016-10	DN2	Jeonmin, Yuseong, Daejeon, Korea	PX313870
		MBC_KIOM-2016-12	DN3	Bisan, Soi, Eumseong, Korea	PX313871
		MBC_KIOM-2016-20	DN4	Bongseo, Gurye, Gurye, Korea	PX313872
		KWJ_KIOM-2016-4	DN5	Sangcheon, Cheongpyeong, Gapyeong, Korea	PX313873
		MBC_KIOM-2016-29	DN6	Jeomchon, Gahoe, Hapcheon, Korea	PX313874

**Table 2:** SCAR primers designed based on species-specific nucleotides within the rDNA-ITS region.

Target Species	Primer	Sequence (5′→3′)	Amplicon (bp)	AT ^a^
*D. sophia*	DS F3	CAT GCA ACA GAA CGG CCA TCT TCT	416	63
	DS R10	GTC ATC AAG AGC TTT CGG ACA A***AC*** A		
*L. densiflorum*	LD F2	GTG GGC CGG TTT CCT AAC AGA ***C***T	202	63
	LD R8	CAG CAC AGC ATT CGC G***TC*** C		
*L. virginicum*	LV F6	GGG CCG GTT TCC TAG ***A***AG A***C***C	493	63
	LV R11	GCA TGT CGG G***T***C GCT C***G***A A		
*E. cheiranthoides*	EC F2	GTC CAG ATC GAG AGT AAA GTT TCG G***G***C	363	67
	EC R8	CGT CCC ATC TCA GCA ATG GAT G***TG*** G		
*E. macilentum*	EM F2	CAT TGT CGA TAC CTG TCC AAA ACA GA***C*** T	486	63
	EM R8	AGA CCA GTT TCC GTC CCA TC***G A***C		
*D. nemorosa*	DN F2	CGT GGT TTC GCG TAT TGC CTT ***T***	433	63
	DN R8	AGA CGT CCT CAG CTC AGA TTT A***CA*** T		

^a^ Annealing temperature (°C). Bold and underlined italic characters denote arbitrary sequences.

## Data Availability

The rDNA-ITS sequences generated in this study are available in the NCBI GenBank database “https://www.ncbi.nlm.nih.gov/ (accessed on 10 September 2025)” under accession numbers PX313848–PX313874.

## Supplementary Materials

The following supporting information can be downloaded at: https://www.mdpi.com/article/10.3390/plants15010073/s1, Figure S1: Multiple alignment of 30 accessions, along with the locations of the designed SCAR primers. Multiple sequence alignments of the rDNA-ITS region of 30 accessions (27 specimens from six species, as three *L. apetalum* accessions registered in NCBI GenBank) were conducted. The colored boxes denote the locations of the SCAR primers designed based on species-specific nucleotides. The direction of the boxes indicates whether the SCAR primers are forward or reverse; Figure S2: Multiple alignment of rDNA-ITS sequences from *D. sophia* accessions originating in Korea and other countries, showing intraspecific conservation of the designed SCAR primer pair (DS F3&R10). A total of 39 *D. sophia* accessions retrieved from NCBI GenBank were analyzed. Blue boxes indicate SCAR primer-binding regions, and grey shading distinguishes the country of origin of each accession; Figure S3: Specimens and seeds of six species used in this study. (A) MBC_KIOM-2016-8 of *D. sophia*; (B) MBC_KIOM-2016-88 #1 of *L. densiflorum*; (C) MBC_KIOM-2016-23 of *L. virginicum*; (D) MBC_KIOM-2016-228 #1 of *E. cheiranthoides*; (E) MBC_KIOM-2016-40 #1 of *E. macilentum*; (F) YSG_KIOM-2016-10 of *D. nemorosa*. (1) specimens; (2) seeds (scale bar = 3mm); Table S1: Genetic diagnosis results for 17 commercial products tested using the SCAR-PCR assay.

## Abbreviations

The following abbreviations are used in this manuscript:

BLAST	Basic Local Alignment Search Tool
ChP 2015	Pharmacopoeia of the People’s Republic of China
DS	Descurainiae Semen
KHP	Korean Herbal Pharmacopoeia
LDS	Lepidii seu Descurainiae Semen
LOD	limit of detection
LS	Lepidii Semen
NCBI	National Centre for Biotechnology Information
NJ	neighbor-joining
rDNA-ITS	ribosomal DNA-internal transcribed spacer
SCAR	sequence-characterized amplified region
SSN	species-specific nucleotide
qPCR	quantitative PCR
