# Supplementary material for: Establishment of ITS-Derived Species-Specific PCR Assay Method for Discriminating Herbal Medicine Descurainiae Semen from Its Commercial Adulterants
Source: Plants (Basel). 2025 Dec 25;15(1):73. doi: 10.3390/plants15010073 (PMC12788001; doi:10.3390/plants15010073)
Supplement: Supplementary file 1 [file plants-15-00073-s001.zip › plants-4050179-supplementary.pdf]

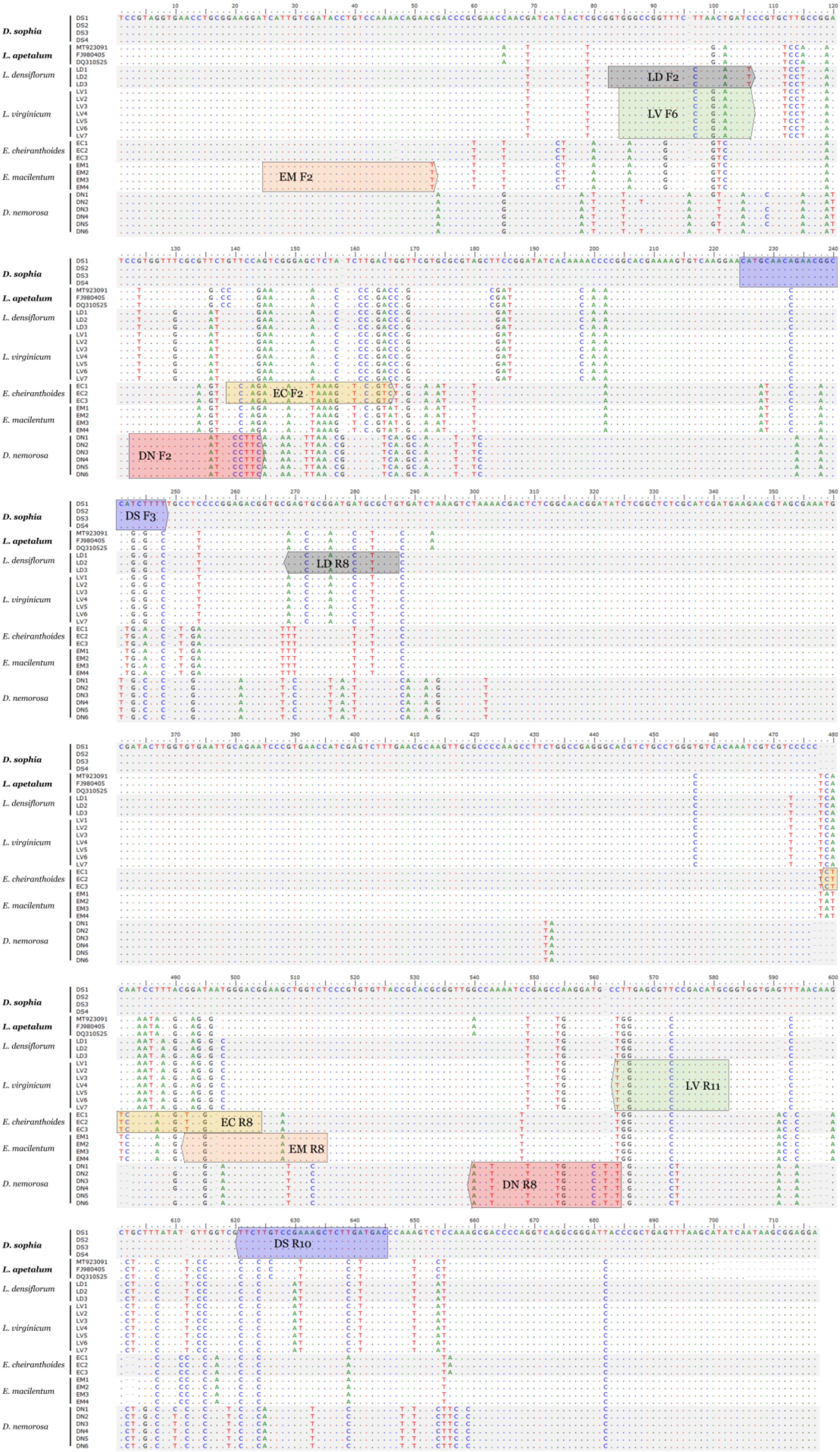

D. sophia
L. apetalum
L. densiflorum
L. virginicum
E. cheiranthoides
E. macilentum
D. nemorosa
DS1
DS2
DS3
DS4
MT923091
FJ980405
DQ310525
LD1
LD2
LD3
LV1
LV2
LV3
LV4
LV5
LV6
LV7
EC1
EC2
EC3
EM1
EM2
EM3
EM4
DN1
DN2
DN3
DN4
DN5
DN6
LD F2
LV F6
EM F2
EC F2
DN F2
DS F3
LD R8
LV R11
EC R8
EM R8
DN R8
DS R10

**Figure S1.** Multiple alignment of 30 accessions, along with the locations of the designed SCAR primers. Multiple sequence alignments of the rDNA-ITS region of 30 accessions (27 specimens from six species, as three *L. apetalum* accessions registered in NCBI GenBank) were conducted. The colored boxes denote the locations of the SCAR primers designed based on species-specific nucleotides. The direction of the boxes indicates whether the SCAR primers are forward or reverse.

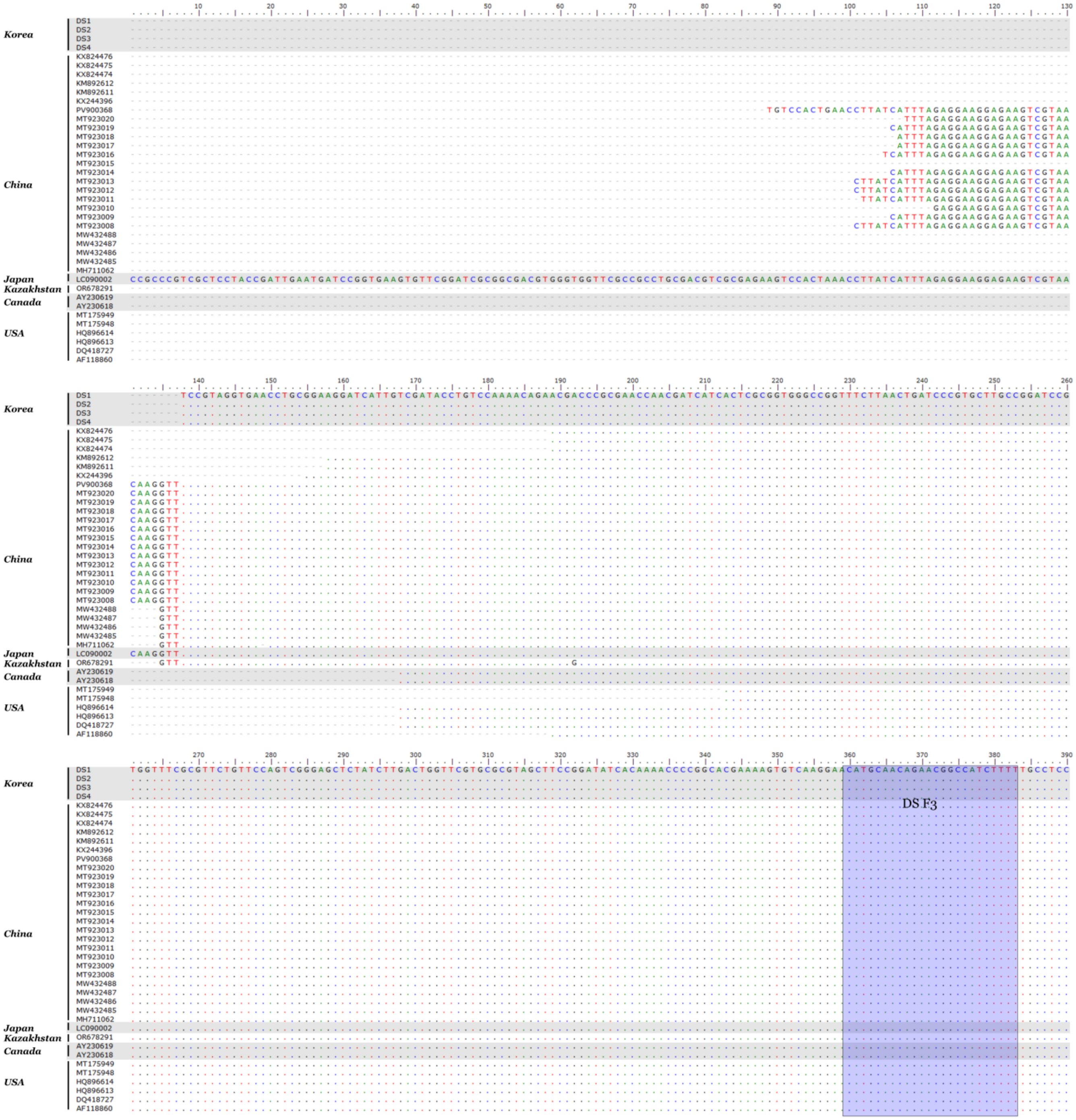


**Figure S2.** Multiple alignment of rDNA-ITS sequences from *D. sophia* accessions originating in Korea and other countries, showing intraspecific conservation of the designed SCAR primer pair (DS F3&R10). A total of 39 *D. sophia* accessions retrieved from NCBI GenBank were analyzed. Blue boxes indicate SCAR primer-binding regions, and grey shading distinguishes the country of origin of each accession.

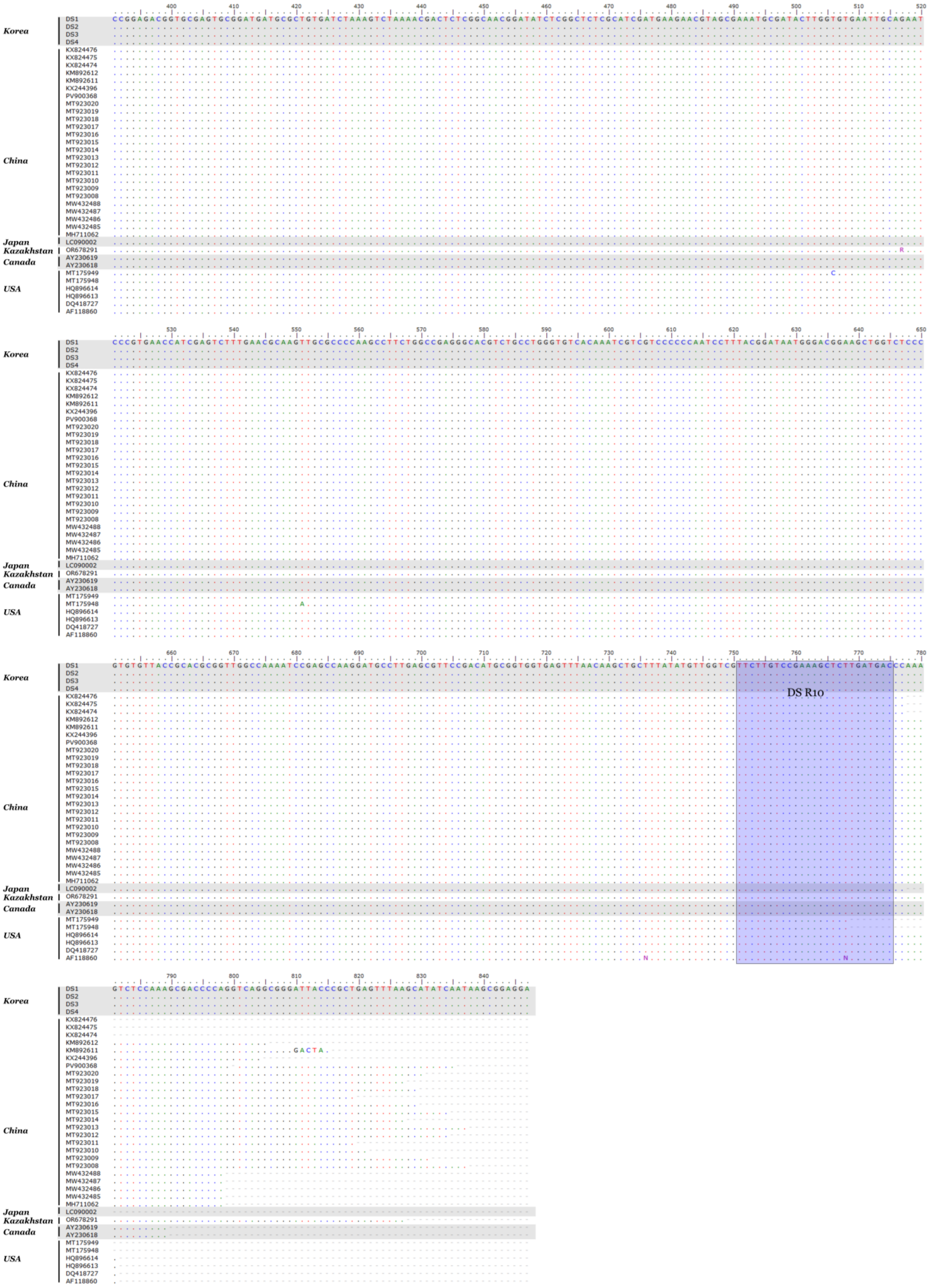


**Figure S2.** (Continued)

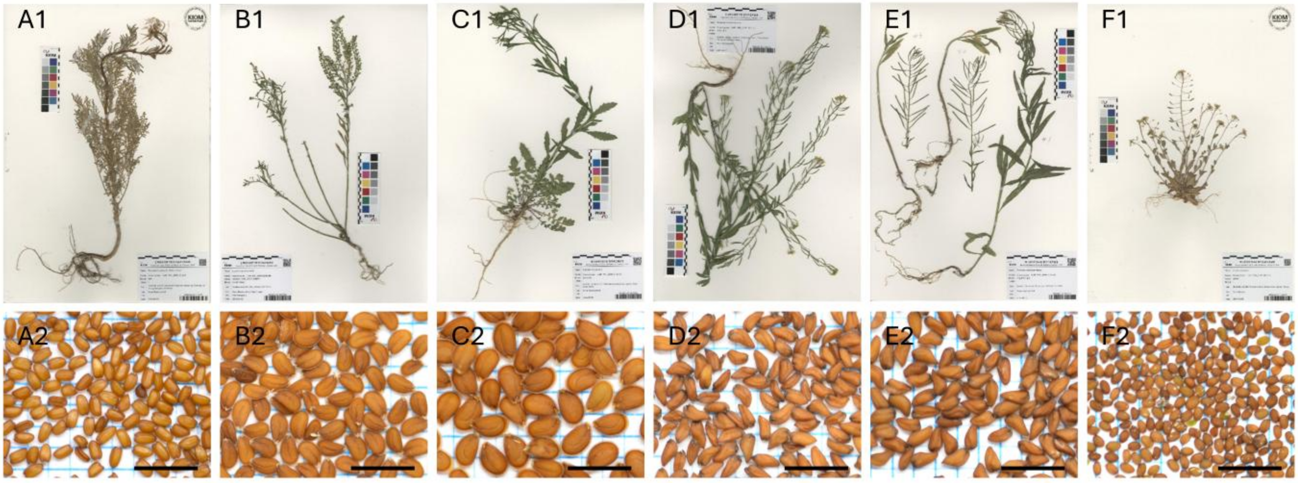


**Figure S3.** Specimens and seeds of six species used in this study. (A) MBC_KIOM-2016-8 of *D. sophia*; (B) MBC_KIOM-2016-88 #1 of *L. densiflorum*; (C) MBC_KIOM-2016-23 of *L. virginicum*; (D) MBC_KIOM-2016-228 #1 of *E. cheiranthoides*; (E) MBC_KIOM-2016-40 #1 of *E. macilentum*; (F) YSG_KIOM-2016-10 of *D. nemorosa*. (1) specimens; (2) seeds (scale bar = 3mm).

**Table S1.** Genetic diagnosis results for 17 commercial products tested using the SCAR-PCR assay

| No. | KIOM resources no. | Purchase year | Manufacturer | Countries of distribution | SCAR-PCR results |
|---|---|---|---|---|---|
| 1 | 2-16-0409 | 2016 | Company A | Korea | *D. sophia* + *E. macilentum* |
| 2 | 2-16-0410 | 2016 | Company A | Korea | *D. sophia* |
| 3 | 2-16-0411 | 2016 | Company B | Korea | *D. sophia* + *E. macilentum* |
| 4 | 2-16-0412 | 2016 | Company B | Korea | *D. sophia* + *E. macilentum* |
| 5 | 2-16-0413 | 2016 | Company C | Korea | *D. sophia* + *E. macilentum* |
| 6 | 2-16-0414 | 2016 | Company C | Korea | *D. sophia* + *E. macilentum* |
| 7 | 2-16-0415 | 2016 | Company D | Korea | *D. sophia* + *E. macilentum* |
| 8 | 2-16-0416 | 2016 | Company D | Korea | *D. sophia* + *E. macilentum* |
| 9 | 2-16-0417 | 2016 | Company E | Korea | *D. sophia* + *E. macilentum* |
| 10 | 2-16-0418 | 2016 | Company F | Korea | *D. sophia* + *E. macilentum* |
| 11 | SO-2016-1 | 2009 | Company G | China | *D. sophia* + *E. macilentum* |
| 12 | SO-2016-2 | 2011 | Company G | China | *D. sophia* + *E. macilentum* |
| 13 | SO-2016-3 | 2012 | Company H | China | *D. sophia* + *E. macilentum* |
| 14 | SO-2016-4 | 2014 | Company G | China | *D. sophia* |
| 15 | SO-2016-5 | 2014 | Company I | China | *D. sophia* + *E. macilentum* |
| 16 | SO-2016-6 | 2014 | Company I | China | *D. sophia* + *E. macilentum* |
| 17 | SO-2016-7 | 2016 | Company J | China | *D. sophia* + *E. macilentum* |
